# Water relations traits of C_4_ grasses depend on phylogenetic lineage, photosynthetic pathway, and habitat water availability

**DOI:** 10.1093/jxb/eru430

**Published:** 2014-12-12

**Authors:** Hui Liu, Colin P. Osborne

**Affiliations:** ^1^Department of Animal and Plant Sciences, University of Sheffield, Sheffield, S10 2TN, UK; ^2^Key Laboratory of Vegetation Restoration and Management of Degraded Ecosystems, South China Botanical Garden, Chinese Academy of Sciences, Xingke Road 723, Tianhe District, Guangzhou 510650, PR China

**Keywords:** C_4_ photosynthesis, C_4_ subtype, drought resistance, leaf hydraulic conductance, osmotic adjustment, phylogeny, Poaceae, stomata, turgor loss point.

## Abstract

The repeated evolution of C_4_ photosynthesis in independent lineages has resulted in distinct biogeographical distributions in different phylogenetic lineages and the variants of C_4_ photosynthesis. However, most previous studies have only considered C_3_/C_4_ differences without considering phylogeny, C_4_ subtype, or habitat characteristics. We hypothesized that independent lineages of C_4_ grasses have structural and physiological traits that adapt them to environments with differing water availability. We measured 40 traits of 33 species from two major C_4_ grass lineages in a common glasshouse environment. Chloridoideae species were shorter, with narrower and longer leaves, smaller but denser stomata, and faster curling leaves than Panicoideae species, but overall differences in leaf hydraulic and gas exchange traits between the two lineages were weak. Chloridoideae species had two different ways to reach higher drought resistance potential than Panicoideae; NAD-ME species used water saving, whereas PCK species used osmotic adjustment. These patterns could be explained by the interactions of lineage×C_4_ subtype and lineage×habitat water availability in affected traits. Specifically, phylogeny tended to have a stronger influence on structural traits, and C_4_ subtype had more important effects on physiological traits. Although hydraulic traits did not differ consistently between lineages, they showed strong covariation and relationships with leaf structure. Thus, phylogenetic lineage, photosynthetic pathway, and adaptation to habitat water availability act together to influence the leaf water relations traits of C_4_ grasses. This work expands our understanding of ecophysiology in major C_4_ grass lineages, with implications for explaining their regional and global distributions in relation to climate.

## Summary

Functional ecological differences between C_4_ grass lineages arise from strong differences in morphological traits but weaker differences in water relations, caused by interactions among phylogeny, C_4_ subtype, and habitat water availability.

## Introduction

The multiple origins of C_4_ photosynthesis in Poaceae represent a classic example of convergent adaptive evolution (Edwards *et al.*, 2010). C_4_ grasses evolved from 22~24 C_3_ lineages under environmental conditions that promote photorespiration, including low atmospheric CO_2_ and high temperatures in open habitats (Sage, 2004; Christin *et al.*, 2008; Osborne and Freckleton, 2009; Edwards *et al.*, 2010; Grass Phylogeny Working Group II, 2012). In these environmental conditions, the C_4_ pathway provides a higher photosynthetic efficiency and maximum carbon-fixation rate (Ehleringer and Björkman, 1977; Sage, 2004), and permits a more efficient hydraulic system (Osborne and Sack, 2012) than the C_3_ type. Both modelling and comparative experiments show hydraulic advantages of C_4_ plants over their C_3_ counterparts. For example, C_4_ grasses can maintain a high photosynthetic rate at lower stomatal conductance (*g*_s_), giving a higher water use efficiency (WUE) than their C_3_ relatives (Osmond *et al.*, 1982; Taylor *et al.*, 2011). C_4_ grasses also have smaller stomata at a given density, resulting in a lower maximum stomatal conductance to water vapour (*g*_max_) (Taylor *et al.*, 2012), as well as a higher proportion of vascular bundle sheath tissue, which may offer hydraulic benefits (Christin *et al.*, 2013; Griffiths *et al.*, 2013). Therefore, during the onset of drought, C_4_ plants can maintain *g*_s_ and photosynthetic rate, as well as leaf water potential (*Ψ*) and hydraulic conductance (Sperry, 2000; Taylor *et al.*, 2011; Osborne and Sack, 2012; Taylor *et al.*, 2014). However, under chronic drought, these advantages may be lost (Ibrahim *et al.*, 2008; Ripley *et al.*, 2010).

Phylogeny and variants of the C_4_ photosynthetic pathway have long been known to have tight associations in Poaceae. Three subtypes within C_4_ grasses are classically defined according to the different enzymes that catalyse the decarboxylation of C_4_ acids: NADP-ME, NAD-ME, and PCK. Previous studies on the biogeography of C_4_ grasses have shown that NAD-ME species occur more in drier places, whereas NADP-ME species prefer wetter areas (Hattersley, 1992; Taub, 2000). However, NAD-ME and NADP-ME species originate almost exclusively from specific lineages, and only PCK species exist across multiple lineages (Sage *et al.*, 1999; Liu *et al.*, 2012). In particular, the predominantly NAD-ME grass lineage Chloridoideae is typically associated with drier environments than the largely NADP-ME lineage Panicoideae (Hartley, 1958*a*, *b*; Hartley and Slater, 1960; Taub, 2000; Edwards *et al.*, 2010; Visser *et al.*, 2012; Visser *et al.*, 2014). Investigation of the interaction between C_4_ subtype and phylogenetic lineage in these taxa has shown that plant traits and habitat water availability (precipitation gradients and habitat wetness) are explained well by phylogeny (Liu *et al.*, 2012). However, these tests only included morphological traits at the genus level, and whether the observed differences in C_4_ subtype distributions are physiologically determined or interact with phylogenetic lineage or habitat water availability remains unclear. More ecophysiological traits related to plant hydraulics are therefore needed to study drought adaptations among C_4_ subtypes.

Most studies on plant hydraulic traits have looked at woody species, especially focusing on the trade-off between hydraulic efficiency and safety (Sack and Holbrook, 2006; Sperry *et al.*, 2008; Meinzer *et al.*, 2010). Reports on the leaf hydraulic conductance (*K*_leaf_) of grasses are rare and only investigate single species (Martre *et al.*, 2001; Holloway-Phillips and Brodribb, 2011). At the leaf scale, plants in dry areas adjust both the concentration of cell osmotic solutes and the elasticity of cell walls to gain greater drought tolerance (Lambers *et al.*, 1998). Pressure–volume (PV) curves describe how *Ψ* changes with the relative water content (RWC) of living leaves during desiccation, providing important hydraulic traits, including the turgor loss point (TLP) (Scholander *et al.*, 1965; Tyree and Richter, 1982; Bartlett *et al.*, 2012). A recent meta-analysis found that osmotic adjustment is more important for setting the TLP than is elasticity, and that less elastic leaves (represented by small cells or high leaf density) contribute to dehydration resistance by maintaining a higher RWC at TLP, thereby reducing the risk of cellular dehydration (Bartlett *et al.*, 2012). Consistent with this observation, C_4_ grasses from dry areas typically have higher leaf dry matter content (LDMC) than those from wet areas (Carmo-Silva *et al.*, 2009). However, whether osmotic or elastic adjustments contribute more to drought adaptations among C_4_ lineages is unknown.

Following up on recent work elucidating the biogeographical and morphological divergences between the two largest subfamilies of C_4_ grasses, Chloridoideae and Panicoideae (Liu *et al.*, 2012), we carried out a species-level study of traits that are involved in plant water relations and drought tolerance in a common glasshouse environment. Our questions included: (i) Does phylogenetic lineage interact with C_4_ subtype or habitat water availability in explaining plant water relations traits? (ii) Do species of Chloridoideae differ from Panicoideae in ecophysiological traits to cope with their naturally drier habitats? Owing to the logistic constraints of working with large numbers of species, we confined our analysis to traits that are constitutively expressed under mesic conditions. Previous work has shown that the hydraulic traits measured for plants growing in a moist environment can predict the range limits of species along aridity gradients (Baltzer *et al.*, 2008; Blackman *et al.*, 2012).

## Materials and methods

### Species sampling

Species were chosen from the two largest C_4_ grass subfamilies, Chloridoideae and Panicoideae, including six main lineages. Cynodonteae, Eragrostideae, and Zoysieae in Chloridoideae; and Paniceae, Paspaleae, and Andropogoneae in Panicoideae (Grass Phylogeny Working Group II, 2012). All three C_4_ subtypes (NADP-ME, NAD-ME, and PCK) were included to balance phylogenetic and photosynthetic groups. It is increasingly recognized that the expression of C_4_ decarboxylases may be flexible within species (Furbank, 2011), and that PCK may be used in a secondary pathway with important physiological functions (Bellasio and Griffiths, 2014; Wang *et al.*, 2014). Our classifications were therefore based upon published evidence about the primary decarboxylase in each case, recognizing that PCK operates in parallel with the other pathways (Wang *et al.*, 2014). As annual and perennial species might have different ecophysiological traits, we selected only perennial species, but one annual PCK species was retained because only PCK species existed in both subfamilies for interaction tests, and few PCK species were available. Nearly 60 candidate species were germinated and finally 33 species were grown on and used in the experiment (Supplementary Table S1). C_4_ subtype was assigned for each species following Sage *et al.* (1999).

### Habitat water availability classification

Three indices were used to measure the habitat water availability for each species: (i) Average regional mean annual temperature (MAT) and mean annual precipitation (MAP). To estimate the realized precipitation niche of each species, geo-referenced species records from the Global Biodiversity Information Facility (GBIF) were collected through GrassPortal (www.grassportal.org). MAT and MAP values from 1961 to 1990 for places that all the samples of one species occurred were averaged as the MAT and MAP value for this species. (ii) Three habitat categories, “wet”, “intermediate”, and “arid”, were assigned based on floras, journal papers, and online herbaria (van Oudtshoorn, 1999; Visser *et al.*, 2012; http://www.efloras.org). The “wet” category included all species described as occurring in wetlands, bogs, swamps, or in/along rivers or other water bodies. The “arid” category included species not belonging to the “wet” category and occurring in well-drained soils described as sandy, stony, gravelly, or rocky. The “intermediate” habitat included all the remaining species, which generally grow in open grasslands or woodlands. (iii) Water score, a value based on the habitat description in floras to quantify habitat water conditions. A numerical score was assigned to each of the habitat types describing water availability, and giving equal weighting to the extremes (Hydrophyte=5, Helophyte=4, Mesophyte=3, Xerophyte=1). This resulted in a continuous sequence of values for each species, which were summarized as a range “water range” and a mean “water score” for each species (Osborne and Freckleton, 2009).

There was a positive relationship between MAP and water score, with Panicoideae in wetter habitats (Supplementary Fig. S1a). The three habitat types were also correlated with MAP, although species of Chloridoideae could also live in wet habitats (e.g. *Spartina pectinata*) and species of Panicoideae in arid habitats (e.g. *Alloteropsis semialata*). Overall, 5, 5, and 2 species of Chloridoideae and 2, 14, and 5 species of Panicoideae were classified into arid, intermediate, and wet habitats, respectively (Supplementary Fig. S1b).

### Plant material, leaf longevity, and glasshouse environment

In May 2009, seeds were surface-sterilized before germination on agar plates in an incubator, and seedlings were then established in small pots of compost (M3, John Innes Seed Compost). Two weeks later, larger seedlings were transferred into 4-litre pots with 50% compost plus 50% silica sand. In total 165 pots, i.e. 5 replicates for each of the 33 species, were arranged in a glasshouse bay in the Arthur Willis Environment Centre (University of Sheffield, UK), following a randomized block design. Plants were watered every day to provide enough water. In the middle of September, supplementary nutrients (Osmocote controlled release plant food) were added to pots. After being transferred into the large pots, one leaf of each plant was tagged and observed every week from 21 July till it died. The process of leaf senescence was recorded as area percentage of dead parts.

From May to October 2009, the growth environment was controlled and recorded via a glasshouse monitoring system and a weather station (DL2e data logger with RHT2nl and QS2 sensors, Delta-T Devices Ltd, Cambridge, UK) at 30min intervals. Day length was set as 14h from June to August, and 17h from August to October. Air temperature was 30/25 °C (day/night), relative humidity was 70%, and the light source was natural light plus four lamps which together delivered 400~1500 µmol m^–2^ s^–1^ in daytime.

### Leaf morphology and stomatal traits

Two mature leaves were taken from each plant: one was saturated with water; the other was used for the curling experiment. Leaves were cut through the sheath under water, transferred into labelled test tubes full of water then placed in a lab sink. The sink was covered by a wet cloth with tap water dripping on it to retain moisture for leaf saturation overnight. The next morning, saturated leaves were cut again at the ligule. Leaf length and width, saturated weight, and leaf volumes (displacement method in a burette) were measured. Next, leaves were scanned on a flatbed scanner (Scanjet 4500c; HP, Berkshire, UK) to obtain the leaf area (ImageJ 1.41, Abramoff, 2004), and then oven-dried (70 °C, 24h) for dry weight. Finally specific leaf area (SLA), LDMC, leaf density, and thickness were calculated.

Imprints with dental putty (President Plus-light body, Coltène/Whaledent Ltd., Burgess Hill, West Sussex, UK) were firstly taken from the lamina of the adaxial and abaxial leaf surfaces, and nail polish impressions were taken from these imprints to make microscope slides. Slides were observed under an inverted microscope equipped with a digital camera (Leica Laborlux S, Wetzlar, Germany) and a computerized image analysis system (Leica Quantimet 500 Q win software). From each peel, we chose three random images as replicates and measured guard cell length (GL), width of the closed guard cell pair (SW), stomatal density (SD) and the stomatal pore area index (SPI), a dimensionless index of stomata pore area per lamina area, where SPI=SD∙GL^2^ (Sack *et al.*, 2003).

The maximum diffusive conductance to water vapour (*g*_max_) is used to estimate transpiration potential at the anatomical level (Brown and Escombe, 1900; Franks and Beerling, 2009).

$$g_{\text{max}}=\frac{d}{v}•D•a_{\text{max}}∕(l+\frac{\pi }{2}\sqrt{a_{\text{max}}∕\pi })$$

where *d* is the diffusivity of water vapour in air at 25 °C (m^2^ s^–1^); *v* is the molar volume of air at 25 °C (m^3^ mol^–1^); *D* is stomatal density (stomata number mm^–2^); *a*_max_ is the maximum area of the open stomatal pore, estimated as *π*∙(*p*/2)^2^ where *p* is stomata pore length [although in Franks and Beerling (2009) *p* was approximated as half of the guard cell length, *p* was measured directly here]; *l* is stomata depth for fully open stomata, approximated as *W*/2; *π* is the geometric constant. We calculated *g*_max_ for both leaf sides, and summed the values to obtain a total leaf value.

### Leaf curling experiment

Leaves were weighed on a four-point balance (AE163; Mettler Toledo Ltd, Leicester, UK) and leaf widths were measured at the widest part. Both weight and width of each leaf were repeatedly recorded for 2h at 15min intervals in a cabinet with constant environment (23~24 °C, RH 60%). Later leaf curling characteristics were recorded and oven-dried leaves were weighed. Owing to the different starting and ending points for different species, all 165 leaf curling models were fitted manually. Leaf width loss rates were slopes of curves of the relative leaf width (% hr^–1^) and absolute leaf width (mm hr^–1^) against time. Leaf weight loss rate (% hr^–1^) was the slope of relative water content against time. During the first hour, leaf stomata were assumed to be still open, but in the next hour most leaves had already curled with stomata closed, and cuticular conductance could therefore be calculated without stomatal transpiration (Boyer *et al.*, 1997). In this study, leaf cuticular conductance (mmol m^–2^ s^–1^) was the slope of the weight loss curve normalized by original leaf area during the second hour. Relative width/weight loss (% hr^–1^) was also calculated as width/weight ratio against time.

### Pressure–volume (PV) curves

Leaves were cut and rehydrated overnight as described above. After measuring the *Ψ* with a pressure chamber (Model 1000 Pressure Chamber Instrument, PMS Instrument Company, USA), the corresponding leaf weight was recorded immediately. The leaves were initially allowed to dry slowly to an equilibrium mass in an airtight box, and then allowed to dry further on the bench, with measurements every 15min in each case. At the end of the experiment, oven-dried leaves were weighed to determine RWC and LDMC, and then PV curves were drawn and analysed. First a straight line was fitted via the stepwise addition of points from the linear portion of the curve at low RWC, where water potential changes only with osmotic pressure. This was used to estimate the response of osmotic potential (*Ψ*_o_) to RWC. The fitted *Ψ*_o_ values were then extrapolated across all RWC values, and subtracted from *Ψ* to obtain the pressure potential (*Ψ*_p_). Secondly, a turgor curve of *Ψ*_p_ against RWC was built by fitting a modified exponential equation (Schulte and Hinckley, 1985), from which the zero intercept is the estimated TLP. Young’s modulus of elasticity (*ε*) was derived from the slope of the moisture release curve between saturation and the TLP (Lenz *et al.*, 2006).

### Leaf hydraulic conductance (*K*_leaf_)

We followed the method of Franks (2006). One mature leaf was cut at the ligule from a well-watered plant. After being weighed on an analytical balance (*W*_1_), it was placed immediately into the pressure chamber. Chamber pressure was increased to the balance pressure of the leaf (*Ψ*_1_) and equilibrated for about 5min. Then chamber pressure was increased rapidly to *Ψ*_2_, with Δ*Ψ* (*Ψ*_2_–*Ψ*_1_) of around 0.5MPa. The sap expressed in the first 10s was removed, using a timer to record the duration, and the final weight of the leaf (*W*_2_) was quickly measured to calculate Δ*W*=*W*_1_ *–W*_2_. Leaves were scanned and oven-dried. *K*_leaf_ was calculated as (Franks, 2006):

$$K_{\text{leaf}}=\Delta W\times {\text{ time}}^{–\text{1}}\times {\text{leaf area}}^{–\text{1}}\times \Delta \Psi^{–\text{1}}$$

### Leaf gas exchange

On sunny days, the youngest mature leaf from a tiller of each plant was chosen and put under high light (1800 µmol m^−2^ s^−1^) to pre-adapt for around 10min. We used an open leaf gas exchange system (LI-6400, LI-COR, Lincoln, NE, USA), setting the conditions of the leaf chamber at: CO_2_ concentration, 400 µmol mol^−1^ (provided by CO_2_ cylinders); leaf block temperature, 30 °C; photosynthetically active radiation (PAR), 2000 µmol m^−2^ s^−1^; flow rate, 400 µmol s^−1^; RH in the sample cell, 70~85 %, to keep the vapour pressure deficit (VPD) in the leaf chamber around 1~1.5 kPa. Maximum net CO_2_ assimilation rate (*A*), stomatal conductance (*g*_s_), intercellular CO_2_ concentration (*C*_i_), and transpiration rate (*E*) were recorded. Instantaneous water use efficiency (WUE_i_) was calculated as *A*/*g*_s_.

### Statistical methods

The distribution of three C_4_ subtypes in the two subfamilies was unbalanced (no NADP-ME species in Chloridoideae, only two NAD-ME species in Panicoideae, more unbalanced at the tribe level). Therefore, to distinguish phylogeny from C_4_ subtype in affecting ecophysiological traits, PCK and non-PCK species were grouped to form a two-factor, complete block design (7 PCK and 5 non-PCK species in Chloridoideae; 7 PCK and 17 non-PCK species in Panicoideae). Generalized linear mixed-effects models (*GLMM*) with phylogeny (subfamily) and photosynthetic type (PCK and non-PCK) were fitted as two fixed factors and species as a random factor; interaction effects were detectable as there were replicates in each unit.

We also tested the effects of phylogeny and habitat water availability on ecophysiological traits using *GLMM*s. MAP, water score, and habitat type were tested separately with phylogeny as two fixed factors, and species as a random factor. Owing to the covariance of MAP, water score and habitat type (Supplementary Fig. S1a), the three models showed the same pattern; therefore only the model with MAP was reported because it had the lowest Akaike information criterion (AIC) value.

### Phylogenetic tree and analysis

As most species were chosen based on published phylogenetic trees of C_4_ lineages, the phylogeny and branch lengths among species were extracted based on the background tree in Christin *et al.* (2008). Six congeners, *Alloteropsis, Brachiaria, Melinis, Urochloa, Chloris*, and *Dactyloctenium*, were used when species sampled in the tree (Christin *et al.*, 2008) were not the same species in this study. However, three species from the genera *Heteropogon, Enneapogon*, and *Fingerhuthia* were not included, so their phylogenetic places were located by their closest genera *Hyparrhenia, Uniola*, and *Eragrostis* (Liu *et al.*, 2012). The final phylogenetic tree clearly represented six lineages (Supplementary Fig. S2).

Phylogenetic analysis reveals the degree of phylogenetic dependence for plant traits. Pagel’s *λ* is based on a Brownian model of trait evolution (Pagel, 1999). The extent to which traits evolve from random drift gives a *λ* value between 0 and 1. The value *λ*=1 implies strong phylogenetic dependence, whereas *λ*=0 indicates no phylogenetic dependence (Freckleton *et al.*, 2002).

### Principal component analysis (PCA) and phylogenetic PCA (PPCA)

Both conventional PCA and PPCA were employed to investigate which traits were most important in distinguishing species. Data were log-transformed (if the original trait values were negative, such as TLP, absolute values were used) to meet the requirement of normal distribution. Conventional PCA was carried out using the R function *princomp*. The same dataset was tested again by phylogenetic PCA (PPCA) such that phylogeny was taken into account (Felsenstein, 1985). A variance–covariance matrix among species was generated by the R function *vcv* in package *ape* and PPCA was carried out using the *phyl.pca* function in package *phytools*.

## Results

### Subfamily comparisons

In the following discussion, we use the term “structural traits” to describe traits that are fixed by development, including morphology, leaf size/mass, and stomatal patterning, and the term “physiological traits” to include those which have dynamic responses to environmental changes, including leaf curling, leaf hydraulics, and leaf gas exchange (Table 1). We use the term “ecophysiological traits” to represent both structural and physiological traits.

**Table 1.** Comparisons of 40 traits between Chloridoideae and Panicoideae, for both total and PCK-only species in the glasshouse experiment Data are means±SEM, sample sizes are shown in the heading. Level of significance for t-tests: * P<0.05; ** P<0.01; *** P<0.001; ns, not significant. Abbreviations: LDMC, leaf dry matter content; SLA, specific leaf area; SD, stomatal density; SPI, stomatal pore index; g_max_, maximum stomatal conductance to water vapour; Ψ_sat_, saturated leaf water potential; Ψ_osat_, saturated leaf osmotic potential; TLP, turgor loss point; ε, Young’s modulus of elasticity; K_leaf_, leaf hydraulic conductance; A, photosynthetic rate; g_s_, stomatal conductance; C_i_, intercellular CO_2_ concentration; E, transpiration rate; WUE_i_, instantaneous water use efficiency; MAT, mean annual temperature; MAP, mean annual precipitation.

	Chloridoideae (total 12 species 7 PCK species)	Panicoideae (total 21 species 7 PCK species)	*P* (total)	*P* (PCK only)
**Morphology and leaf longevity**				
Culm height (cm)	55±5.5	91±6.2	***	ns
Leaf length (cm)	41±2.4	34±1.4	*	***
Leaf width (mm)	6±0.3	11±0.4	***	***
Leaf area (cm^2^)	22±1.6	28±1.7	**	ns
Leaf volume (cm^3^)	0.5±0.04	0.6±0.04	*	*
Leaf dry weight (mg)	69±8.0	78±5.3	ns	*
Seed size (mm^2^)	.88±.15	2.11±.12	***	**
Leaf longevity (days)	53±2.1	57±2.4	ns	ns
**Leaf structure**				
LDMC (%)	17±0.6	17±0.4	ns	ns
SLA (m^2^ kg^–1^)	45±2.1	40±1.0	*	ns
Leaf density (g cm^–3^)	0.14±0.006	0.14±0.005	ns	ns
Leaf thickness (mm)	0.19±0.011	0.21±0.005	ns	ns
**Stomatal traits**				
Guard cell length (µm)	24±1.0	32±0.9	***	***
Stomatal width (µm)	15±0.4	18±0.4	***	ns
Stomatal pore length (µm)	12±0.5	18±0.7	***	***
Abaxial SD (mm^–2^)	223±18.3	136±6.9	***	***
Adaxial SD (mm^–2^)	147±16.3	105±7.7	*	ns
Abaxial SPI (dimensionless)	10.9±0.56	13.4±0.54	**	ns
Adaxial SPI (dimensionless)	7.5±0.74	9.1±0.52	ns	ns
*g*_max_ (mol m^–2^ s^–1^)	5.0±0.24	4.5±0.16	ns	ns
**Leaf curling**				
**Leaf curling rate**				
Relative width loss (% hr ^–1^)	77±10	37±3	***	*
Absolute width loss (mm hr ^–1^)	2.6±0.26	2.5±0.21	ns	ns
**Leaf weight loss rate**				
Relative weight loss (% hr ^–1^)	14±0.4	14±0.5	ns	*
Leaf cuticular conductance (mmol m^–2^ s^–1^)	0.27±0.019	0.29±0.015	*	ns
Relative width/weight ratio loss (% hr ^–1^)	4.88±0.51	2.53±0.24	**	ns
**Leaf hydraulic traits**				
*Ψ*_sat_ (MPa)	–0.20±0.02	–0.16±0.01	*	ns
*Ψ*_osat_ (MPa)	–1.1±0.04	–1.0±0.02	ns	**
TLP (MPa)	–1.2±0.04	–1.1±0.03	ns	***
*ε* (MPa)	0.12±0.009	0.11±0.004	ns	*
*Ψ* (MPa)	–0.54±0.034	–0.45±0.020	*	ns
*K*_leaf_ (mmol m^–2^ s^–1^ MPa^–1^)	17±0.9	19±0.7	*	**
**Leaf gas exchange**				
*A* (µmol CO_2_ m^–2^ s^–1^)	18±1.1	17±0.6	ns	ns
*g*_s_ (mol H_2_O m^–2^ s^–1^)	0.16±0.012	0.17±0.008	ns	*
*C*_i_ (µmol CO_2_ mol^–1^)	190±5.5	196±4.2	ns	ns
*E* (mmol H_2_O m^–2^ s^–1^)	3±0.2	3±0.1	ns	ns
WUE_i_ (A/*g*_s_)	117±3.3	111±2.5	ns	ns
**Habitat traits**				
Water range	2.0±0.6	2.1±0.8	ns	ns
Water score	1.95±0.56	2.83±0.56	***	**
MAT (°C)	17.8±1.6	19.9±0.8	ns	ns
MAP (mm)	764±98	1090±75	*	ns

The two taxonomic groups differed significantly in their structural traits. Chloridoideae species were shorter plants with smaller seeds, but with longer and much narrower leaves than Panicoideae, which led to a smaller area and volume for individual leaves (Table 1, all *P*<0.05). The dry weight of individual leaves was similar in the two subfamilies, which lead to indistinguishable leaf dry matter content (LDMC), leaf density and thickness, but 11% greater specific leaf area (SLA) in Chloridoideae than Panicoideae. Chloridoideae showed distinctively smaller stomatal length and width, but higher stomatal density than Panicoideae. Stomatal size was similar between the two leaf sides, but stomatal density and SPI were much higher for the abaxial than adaxial surface (*P*<0.05). Chloridoideae had smaller abaxial SPI (*P*<0.01) than Panicoideae (Table 1). The two subfamilies also had similar leaf longevity of around 55 days; although each species had a different leaf life span and mortality rate, the overall duration of senescence was around 40% of the leaf lifespan.

Several physiological traits differed among the taxonomic groups. Leaves of Chloridoideae curled and lost water at a similar absolute rate to those of Panicoideae after excision. However, owing to narrower absolute leaf widths, relative width loss of Chloridoideae was much faster, and rolling was completed in a shorter time. After stomata closed, leaf cuticular conductance was slightly smaller for Chloridoideae than Panicoideae (Table 1). Chloridoideae also operated at a more negative leaf water potential in ambient conditions (*Ψ*) and at saturation (*Ψ*_sat_) than Panicoideae. The *K*_leaf_ of Chloridoideae was lower than that of Panicoideae. For leaf gas exchange, the two subfamilies showed no significant differences. Finally, habitat water score and MAP of Chloridoideae species were lower than those of Panicoideae (Table 1).

When only PCK species were included, results of *t*-tests on some traits between the two subfamilies changed (Table 1). Culm height, leaf area, and SLA were no longer significantly different, and nor were stomatal width, adaxial stomatal density, SPI, leaf cuticular conductance, *Ψ*, *Ψ*_sat_, and MAP. However, leaf dry weight, relative weight loss, *Ψ*_osat_, TLP, *ε*, and *g*_s_ now differed significantly (Table 1).

### Phylogenetic signals

Most traits did not show phylogenetic dependence (Table 2A). None of the structural traits, except leaf width and seed size, showed phylogenetic signals, and neither did any other leaf structural traits including LDMC (all *λ* values<0.06, with *P*>0.05 for *λ=*0). However, most of the stomatal traits showed high *λ* values, with the exception of adaxial stomatal density. The derived stomatal indices SPI and *g*_max_ had no phylogenetic signals. None of the *λ* values of parameters involved in the leaf curling, leaf longevity, hydraulic measurements and leaf gas exchange, were significantly different from zero, except leaf relative width and width/weight loss. For habitat traits, strong phylogenetic signals were found for water score and MAT, but there were no signals for water range and MAP (Table 2A).

**Table 2.** Results for (A) phylogenetic tests, (B) generalized linear mixed-effects models (GLMM) for phylogeny×photosynthetic type, and (C) GLMM for phylogeny×habitat water availability (A) Estimated λ values for 40 indices of the 33 grass species (*N*). (B) GLMM with phylogeny (S, subfamily) and photosynthetic type (PT, PCK, and non-PCK) as two fixed factors, species as a random effect. (C) GLMM with phylogeny (S, subfamily) and habitat water availability (MAP, mean annual precipitation) as two fixed factors, species as a random effect. Sample size (n), λ values, P values for phylogenetic tests; Total number of individuals sampled (n), F values, d.f. (for each factor), and P values for GLMM are reported. Level of significance: * P<0.05; ** P<0.01; *** P<0.001; ns, not significant. Significant results are in bold.

**Ecophysiological trait**	*N*	(A) Phylogenetic test			*n*	(B) *GLMM* of C_4_ subtype			(C) *GLMM* of habitat water		
		***λ***	*P* (*λ*=0)	*P* (*λ*=1)		S (1, 29)	PT (1, 29)	S×PT (1, 29)	S (1, 29)	MAP (1, 29)	S×MAP (1, 29)
**Morphology and leaf longevity**											
Culm height	33	0.03	ns	**	165	3.12*	.05	.99	2.46	.01	.38
Leaf length	33	0.00	ns	***	224	4.65*	3.25	1.22	1.35*	.02	.71
Leaf width	33	**0.28**	*	***	224	10.22**	.20	.32	7.63*	.28	.46
Leaf area	33	0.00	ns	***	223	1.39	.04	2.16	.85	.36	.12
Leaf volume	33	0.00	ns	***	223	1.06	.03	4.81*	.49	.43	.62
Leaf dry weight	33	0.00	ns	***	223	.31	.25	4.38*	.14	1.23	1.56
Seed size	33	**0.25**	*	***	165	56.24***	2.75	1.12	58.85***	7.73**	8.17**
Leaf longevity (days)	33	0.00	ns	**	163	.24	.61	.82	.34	1.37	.25
**Leaf structure**											
LDMC	33	0.00	ns	***	222	.00	.44	.16	.02	.63	4.67*
SLA	33	0.00	ns	***	214	1.27	2.02	2.88	.22	.40	4.96*
Leaf density	33	0.00	ns	***	217	.02	1.00	.66	.00	1.53	5.14*
Leaf thickness	33	0.00	ns	***	222	.92	.06	3.96	.31	.09	.30
**Stomatal traits**											
Guard cell length	33	**0.71**	***	**	99	18.89***	1.63	.23	16.63***	1.09	1.64
Stomatal width	33	**0.62**	*	**	99	9.71**	3.37	1.13	7.29**	.31	.19
Stomatal pore length	33	**0.58**	**	***	99	15.14***	.69	.22	14.18***	.91	2.20
Abaxial SD	33	**0.93**	***	ns	92	13.17**	4.70*	3.89	14.01***	.26	3.10
Adaxial SD	33	0.00	ns	***	99	8.76*	1.27	2.07	3.00	.00	2.65
Abaxial SPI	33	0.08	ns	***	92	6.08*	1.30	8.33**	3.07*	.20	.23
Adaxial SPI	33	0.01	ns	***	99	1.07	.41	1.18	.68	.05	.23
*g*_max_	33	0.00	ns	ns	99	1.37	4.22*	.01	3.27	.02	7.38
**Leaf curling**											
**Leaf curling rate**											
Relative width loss	33	**0.52**	**	***	163	14.97***	.01	.15	13.12**	1.94	4.17
Absolute width loss	33	0.00	ns	***	163	.02	1.53	.00	.21	.12	1.32
**Leaf weight loss rate**											
Relative weight loss	33	0.00	ns	**	163	.00	.32	2.48	.15	.00	.27
Leaf cuticular conductance	33	0.00	ns	*	163	1.42	.02	.04	.61	.28	.73
Relative width/weight ratio loss	33	**0.36**	ns	***	163	5.79*	.40	.01	3.21	1.58	1.53
**Leaf hydraulic traits**											
*Ψ*_sat_	33	0.03	ns	***	162	3.46*	.01	.03	3.28	2.88	7.21*
*Ψ*_osat_	33	0.00	ns	**	162	1.10	1.03	4.81*	2.31	.62	3.48
TLP	33	0.00	ns	*	162	1.01	1.28	5.06*	2.48	.56	2.91
*ε*	33	0.00	ns	**	162	1.11	1.55	2.66*	2.74	.95	7.82**
*Ψ*	33	0.01	ns	**	196	3.08*	1.32	.76	2.29	.06	.62
*K*_leaf_	33	0.00	ns	***	165	1.37	.03	2.12*	.64	.33	1.36
**Leaf gas exchange**											
*A*	33	0.00	ns	***	140	.13	.27	3.26	.31	.10	.29
*g*_s_	33	0.00	ns	***	140	.11	.15	1.48	.05	.02	.01
*C*_i_	33	0.00	ns	***	137	.34	.00	.09	.02	.01	.46
*E*	33	0.00	ns	***	140	.05	.06	2.94	.41	.20	.09
WUE_i_	33	0.00	ns	***	140	1.29	.01	.02	.23	.03	.33
**Habitat traits**											
Water range	33	0.00	ns	***	33	.27	.00	.00	1.06	1.68	1.54
Water score	33	**0.55**	*	ns	33	17.28**	.82	.21	20.53***	20.66***	11.43**
MAT	28	**0.90**	*	ns	28	1.92	2.96	.08	.07	23.89***	10.13**
MAP	28	0.22	ns	***	28	6.71*	.47	.60	**–**	**–**	–

### PCA and PPCA results

**Fig. 1.**
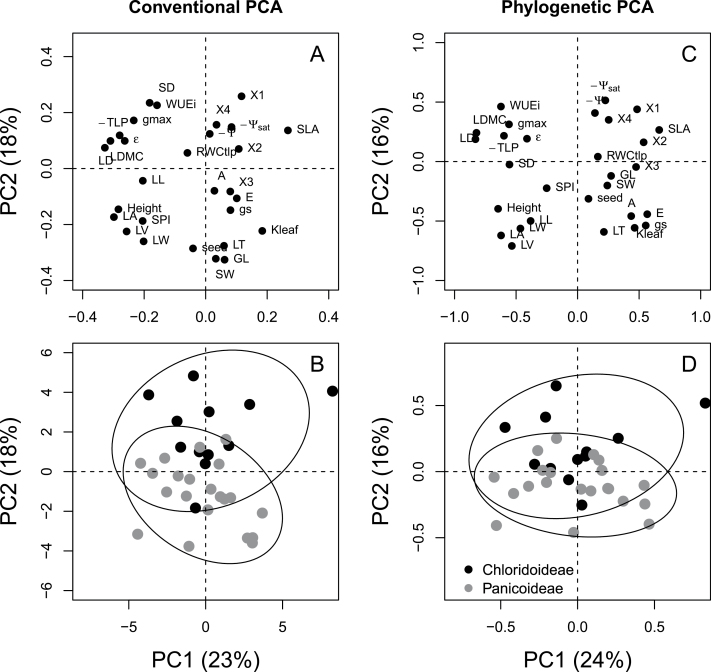
Conventional principal component analysis (PCA) and phylogenetic PCA (PPCA) for the first two principal components (PC) based on 29 plant traits of 33 C_4_ grasses. (A, C) PC loadings and (B, D) species scores with Chloridoideae (black) and Panicoideae (grey) circled. The percentages of variance explained by the first two PCs are in the axis labels. See Supplementary Table S2 for PC loadings, Table 1 for trait abbreviations, with the addition of: LD, leaf density; LA, leaf area; GL, guard cell length; SW, stomatal width; LT, leaf thickness; LW, leaf width; LV, leaf volume; X1, relative width loss; X2, relative weight loss; X3, leaf cuticular conductance; X4, relative width/weight ratio loss.

In the conventional PCA, the first two axes explained 23% and 18% of total variation, respectively (Fig. 1A, B; Supplementary Table S2). Leaf structural traits (density, LDMC, area), culm height, and TLP were on the negative side of PC1, whereas SLA was on the positive side of PC1. Guard cell length, stomatal width, seed size, and leaf thickness and width were loaded on the negative side of PC2, whereas stomatal density and relative width loss during leaf curling were on the positive side of PC2 (Fig. 1A). PCA could not distinguish species between Chloridoideae and Panicoideae, or species from three C_4_ subtypes along PC1. However, the two subfamilies were separated along PC2, showing larger stomata and slower leaf curling rates for Panicoideae species as in Fig. 1A (Fig. 1B).

After accounting for the phylogenetic background, PPCA showed different patterns and explained the total variation as 24% and 16% for PC1 and PC2, respectively (Fig. 1C, D; Supplementary Table S2b). As with the conventional PCA, leaf structural traits and TLP loaded on PC1, but stomatal traits, seed size and leaf curling traits were no longer on PC2, with the exception of leaf size traits (Fig. 1C). Instead, leaf hydraulic traits (*K*_leaf_, *g*_s,_ and *Ψ*_sat_) emerged along PC2 in separating the two subfamilies, although the extent of separation was weaker in PPCA (Fig. 1D).

### Phylogeny interacted with C_4_ subtype and habitat water availability

**Fig. 2.**
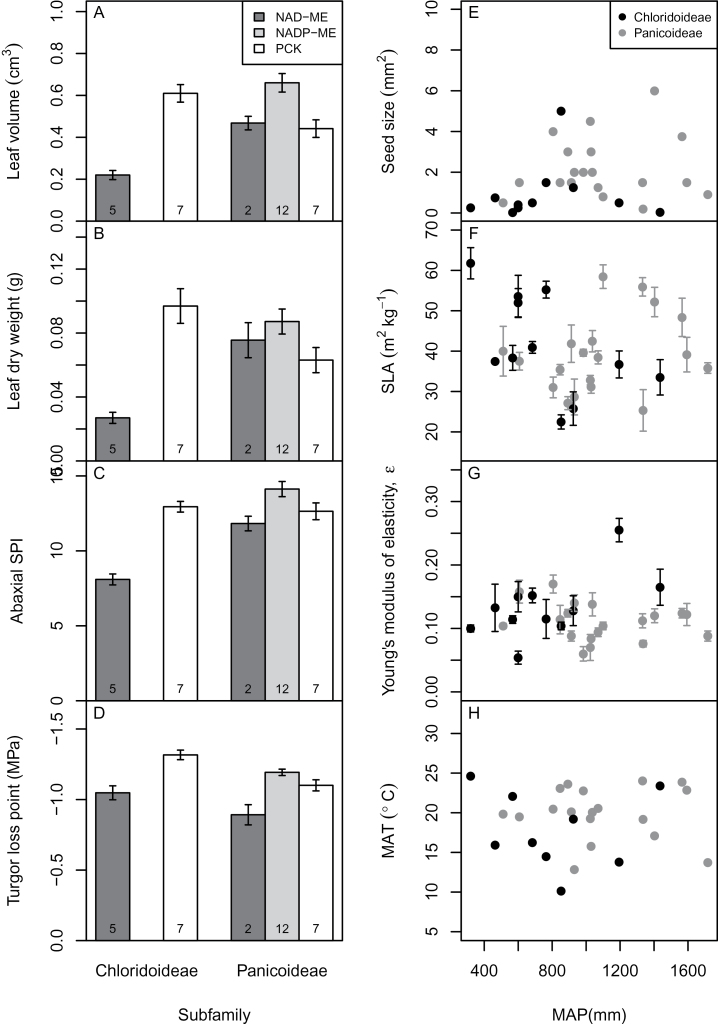
Comparisons of trait values with significant two-factor interactions. (A–D) Subfamily×C_4_ subtype and (E–H) subfamily×habitat type. Traits are selected according to *GLMM* results (Table 2b–c). Species numbers are shown under each bar.

Phylogeny and C_4_ subtype affected ecophysiological traits differently. Subfamily divergences were found for culm height, leaf length and width, seed size, stomatal size-related traits, leaf relative width loss, *Ψ*_sat_, *Ψ*, water score, and MAP. Meanwhile, only *g*_max_ was affected by C_4_ subtype (Table 2B). Although several indices showed significant interactions, most of them were affected by neither subfamily nor C_4_ subtype (Table 2B). Compared with the exploratory two-way ANOVA results (data not shown), species as a random factor in *GLMM* reduced detectable effects from fixed factors or their interaction, indicating that inter-specific differences were an important source of variance. Traits with significant C_4_ subtype or interaction effects were plotted in Fig. 2A–D. Within Chloridoideae, PCK species always had higher values than NAD-ME species, whereas within Panicoideae, there were no general patterns among three C_4_ subtypes, but the value of PCK species was the main driver for an interaction.

Habitat water availability also interacted with phylogeny, although most traits were affected by neither of the two factors (Table 2C). MAP alone had no effects but it interacted with subfamily for several traits, including seed size, LDMC, SLA, leaf density, *Ψ*_sat_, *ε*, water score, and MAT. Traits with significant interaction effects were further investigated (Fig. 2E–H). Within Chloridoideae, particular species had extreme values; for example, *Zoysia japonica* lives in wet habitats and has large seeds. High average SLA values of Chloridoideae were driven by two species (*Sporobolus nebulosus* and *Dactyloctenium scindicum*). Two species in Chloridoideae, *Zoysia japonica* and *Chloris elata*, had extremely high *ε* values. The MAT of the realized niche for each species showed no clear pattern with MAP.

### Leaf water relations traits

**Fig. 3.**
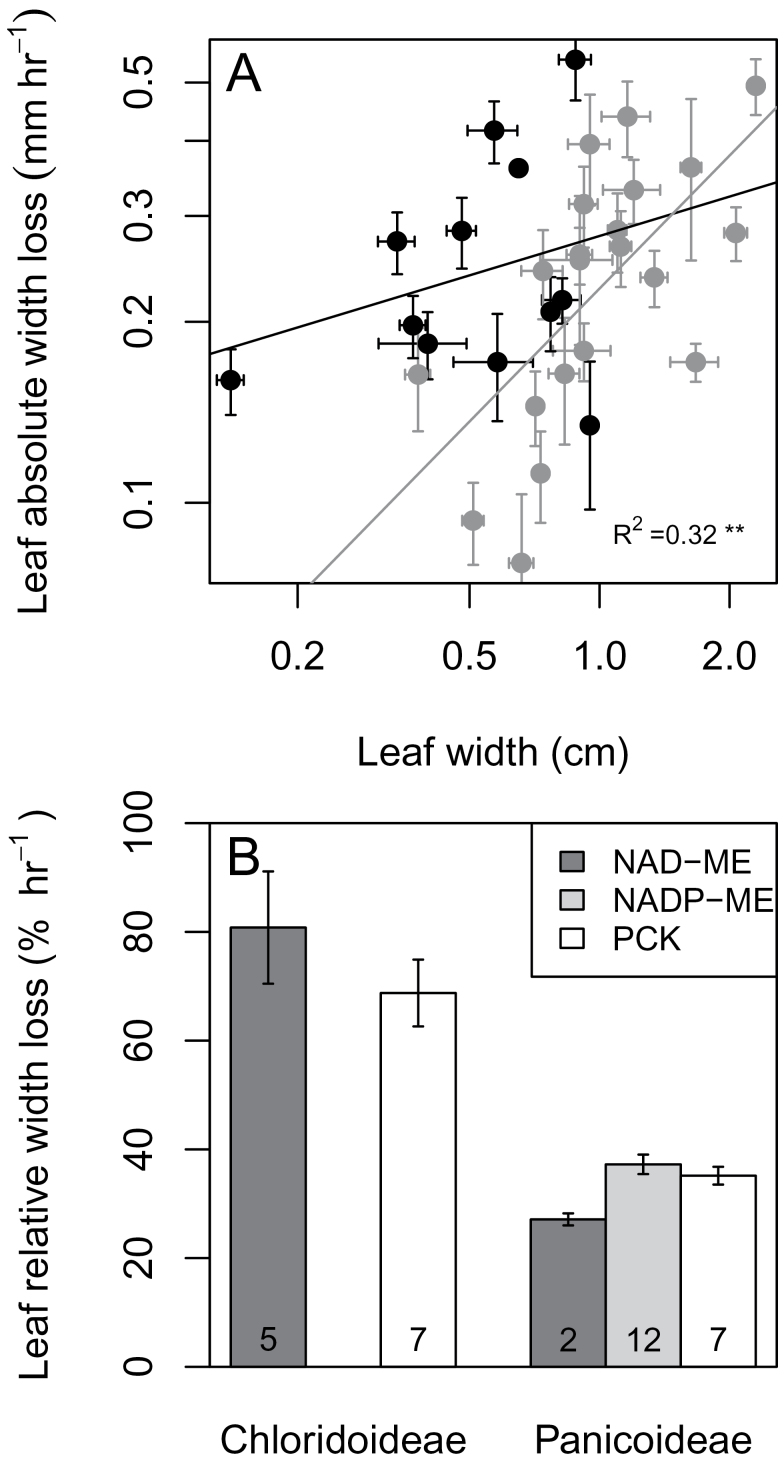
Leaf rolling in response to water loss. (A) Absolute leaf width loss against leaf width. (B) Relative leaf width loss for subfamilies and C_4_ subtypes. In A, subfamilies are Chloridoideae (black) and Panicoideae (grey), and each point is a species with means±SE (*n*=6). In B, species numbers are shown under each bar.

The comparison of leaf curling showed that the wider a leaf was, the faster the absolute rate of curling, with subfamily as an important factor in explaining variance (Fig. 3A). Although the two subfamilies had similar absolute width loss rates, the time to achieve curling end points was much faster for Chloridoideae species, with an extreme species, *Sporobolus nebulosus* that could quickly curl in 10min (Fig. 3B). On the other hand, leaf cuticular conductance and relative weight loss showed no differences among species.

**Fig. 4.**
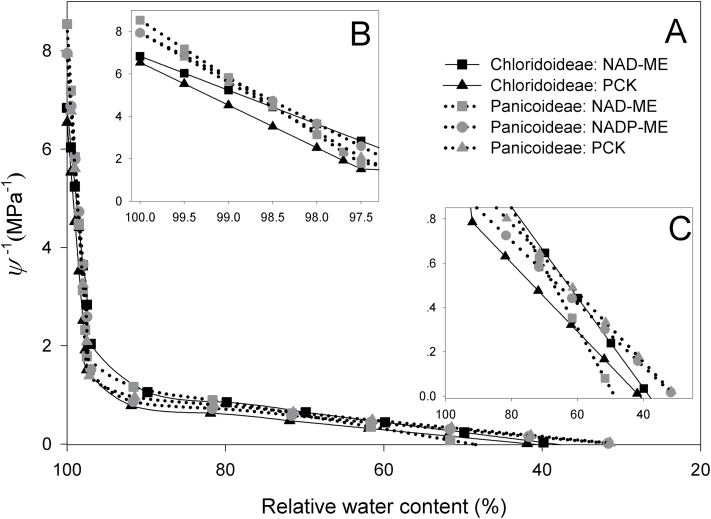
Physiological responses to leaf water loss. Pressure–volume (PV) curves of different subfamily and C_4_ subtype groups. (A) The entire PV curve; (B) a magnified view of turgor loss phase and (C) a magnified view of osmotic loss phase. Curves are modelled on average values of species in each group.

As subfamily and C_4_ subtype interactively affected PV curve parameters (Table 2B), they were analysed separately (Fig. 4A). In the turgor loss phase, NAD-ME species in Chloridoideae had the most elastic leaves (the shallowest slope indicates the smallest *ε*), whereas PCK species in Chloridoideae had the least elastic leaves. These two groups of Chloridoideae species also had more negative *Ψ*_sat_ (i.e. lower *y* intercepts) than Panicoideae species (Fig. 4B). In the osmotic phase, NAD-ME species from both subfamilies showed the two steepest slopes, indicating less negative *Ψ*_osat_ and TLP, whereas PCK species in Chloridoideae had the most negative *Ψ*_osat_ and TLP (Fig. 4C; Supplementary Table S3).

**Fig. 5.**
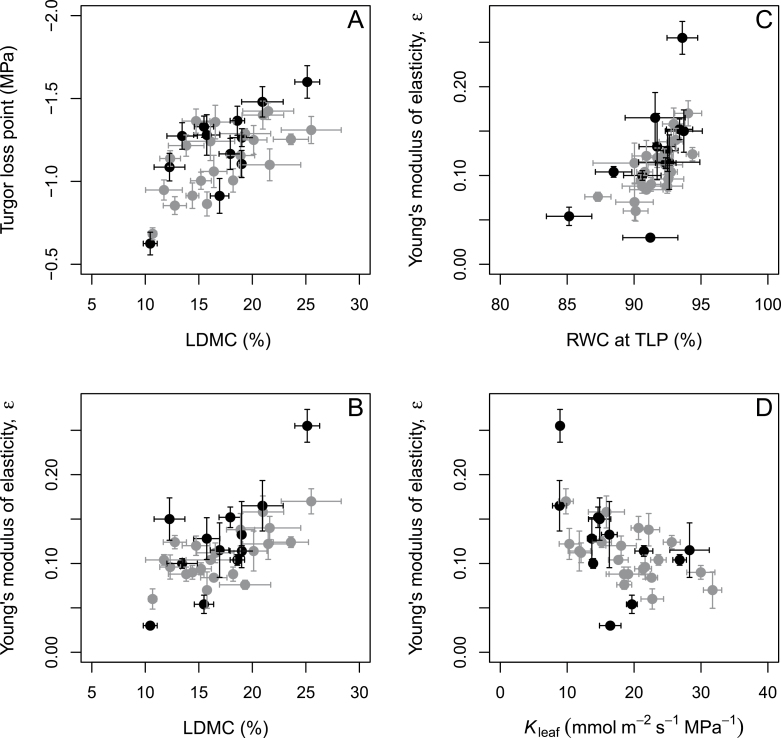
Correlations among leaf hydraulic traits. (A) TLP with LDMC, Young’s modulus of elasticity (*ε*) with (B) LDMC, (C) RWC at TLP, and (D) leaf hydraulic conductance (*K*_leaf_).

There were relationships among different hydraulic and structural traits. Both TLP and *ε* increased with LDMC, suggesting that greater LDMC was associated with less elasticity and a more negative TLP (Fig. 5A, B). The *ε* was positively related with RWC at the TLP, meaning that a steeper initial fall in pressure potential with RWC led to higher RWC at the TLP (Fig. 5C). *ε* was negatively related with *K*_leaf_, such that that the most elastic leaves also had the highest hydraulic conductance (Fig. 5D). Other leaf traits such as SLA, leaf thickness, and volume were also explored in the tests, but only LDMC showed significant relationships with hydraulic traits.

## Discussion

### Phylogeny interacts with photosynthetic type and habitat water availability

Phylogenetic patterns have previously been recognized in the distributions of C_3_ and C_4_ photosynthetic types (Edwards *et al.*, 2010), C_4_ biochemical subtypes (Taub, 2000), and habitat water gradients (Osborne and Freckleton, 2009) among species. However, this is the first study to investigate the ecophysiological adaptations associated with these patterns. We found that phylogeny interacted with both photosynthetic type and water availability for some structural and most hydraulic traits (Fig. 2; Table 2B, 2C).

Previous studies comparing ecophysiological traits between NAD-ME and NADP-ME species have implied that there may be an interaction between phylogeny and C_4_ subtype, but none of them had sufficient statistical power to test it directly (Ghannoum *et al.*, 2002; Taylor *et al.*, 2010). Our results provided solid evidence that both phylogeny and C_4_ subtype are associated with ecophysiological trait variation. Specifically, phylogeny tended to have a greater influence on structural traits (Table 2A), whereas C_4_ subtype tended to be more related with physiological traits, and their interactions especially affected leaf hydraulic traits (Table 2B). One explanation for this pattern may be that structural traits from distant lineages are less labile during evolution than physiological traits (Ackerly and Donoghue, 1998). In contrast, leaf physiological traits depend more on instantaneous responses like stomatal behaviour and enzyme activities, which are likely to be influenced more by C_4_ subtypes (Ghannoum, 2009). Another possible explanation for weak phylogenetic signals in physiological traits is that species diversity in this study was limited, and phylogenetic signals become harder to detect at a small phylogenetic scale (Cavender-Bares *et al.*, 2009).

In the *GLMM*s we classified photosynthetic type into PCK/non-PCK groups, as only PCK species occur in multiple lineages across both subfamilies. The interaction between PCK/non-PCK groups and subfamily indicated the possibility that PCK may operate plastically in otherwise NADP-ME or NAD-ME species, which corresponds to recent biochemical and molecular studies finding the co-existence of PCK activity in NADP-ME or NAD-ME species (Bräutigam *et al.*, 2014; Wang *et al.*, 2014). Therefore, whether ecophysiological traits of plants reflects biochemical differences needs more study, and care should be taken in future comparative experiments based on classical C_4_ subtypes, especially when they have independent origins.

Habitat water availability has been an important selection force in C_4_ grass evolution (Osborne and Freckleton, 2009), and is an important determinant of the phylogenetic composition of C_4_ grass communities (Visser *et al.*, 2012). The interaction between water availability and phylogeny in explaining ecophysiological traits thus suggests different adaptation strategies in different lineages, particularly represented by leaf water relations traits (Table 2C). For example, within Chloridoideae, *Zoysia japonica* lives in wet habitats with large seeds and high *ε* values, and *Sporobolus nebulosus* has a high SLA (Fig. 2E–H), which indicates that not all Chloridoideae species live in dry areas with drought tolerant plant traits.

### Distinct structural but subtle physiological differences

Most Chloridoideae species occur in drier habitats, and have leaf structures with more drought tolerance potential, but do not show significant hydraulic advantages over Panicoideae except in relative curling rate, *Ψ*, *Ψ*_sat_, and *K*_leaf_ (Table 1). However, the difference between PCA and PPCA indicated that, after considering phylogenetic relationships, some leaf hydraulic traits (*K*_leaf_, *g*_s,_ and *Ψ*_sat_) did have the potential to distinguish the two subfamilies (Fig. 1B–D), despite the weak phylogenetic signals of most physiological traits (Table 2A).

Structurally, smaller but denser stomata, as in Chloridoideae compared with Panicoideae, are more rapidly controlled during short-term water stress (Franks and Farquhar, 2007). Longer and much narrower leaves of Chloridoideae are also directly related with a faster relative curling rate. Quick-to-achieve leaf curling end points are ecologically meaningful as they offer an effective means of saving water during drought, and curling is found in many species of dry habitats (Grammatikopoulos and Manetas, 1994). Chloridoideae also had higher SLA, which was linked with lower leaf cuticular conductance and *K*_leaf_, implying a higher internal resistance of leaves (Sack *et al.*, 2013).

PV curves detected two strategies for species in Chloridoideae to reach a higher potential drought resistance than Panicoideae (Fig. 4; Supplementary Table S3). Within Chloridoideae, NAD-ME species had highly elastic leaves (lower *ε*), giving the largest capacity to deviate from an ideal osmotic system (lower *Ψ*_sat_ and RWC at TLP), which may buffer transient changes in transpiration and contribute to water storage for survival after stomata close (Sack *et al.*, 2003; Bartlett *et al.*, 2012). These traits imply a drought avoidance strategy in NAD-ME species of Chloridoideae. In contrast, PCK species in Chloridoideae achieved low osmotic potentials (lower *Ψ*_osat_ and TLP), implying high solute concentrations in cells for either osmo-protection or antioxidant defence (Gullo and Salleo, 1988; Carmo-Silva *et al.*, 2009), and a drought tolerance strategy. In this study, lower *ε* and more negative *Ψ*_osat_ did not occur together in the same species, as they contributed to drought strategies differently, i.e. *Ψ*_osat_ influenced the TLP, whereas *ε* was associated more with RWC at the TLP (Bartlett *et al.*, 2012).

We also uncovered several ecophysiological patterns by integrating water relations traits: (i) leaf stomatal and cuticular conductances to water vapour, and hydraulic conductance to liquid water were lower in Chloridoideae than Panicoideae owing to the linkages between hydraulics, transpiration, and stomatal control (Osborne and Sack, 2012). However, these relationships are weakened by their semi-independent responses to irradiance and dehydration (Guyot *et al.*, 2012). (ii) Among a series of leaf structural traits, only LDMC showed significant relationships with hydraulic traits such as TLP and *K*_leaf_ (Fig. 5), suggesting that some leaf economic spectrum traits were linked with hydraulic traits. (iii) We did not find any relationships between leaf longevity and other ecophysiological traits in C_4_ grasses, although leaf longevity is typically correlated with leaf construction and physiological traits (Wright *et al.*, 2004) and *K*_leaf_ (Simonin *et al.*, 2012) in most dicots. The lack of a relationship may arise because grass leaves all turn over quickly, which means there is little interspecific variation among the species investigated.

The lack of significant physiological advantages for Chloridoideae species in this study might also arise from the differences between glasshouse and field conditions. For example, a humid (RH 70%) environment and no drought stress might limit the hydraulic advantages of dumbbell shaped stomata under high humidity (Franks and Farquhar, 2007). Furthermore, some C_4_ species in drier places might use a drought escape strategy by growing in the rainy and hot summer to avoid drought or frost (Lambers *et al.*, 1998; Ibrahim *et al.*, 2008), which could also confound direct comparisons obtained in a glasshouse.

## Conclusions

Phylogenetic divergences within C_4_ grasses have given rise to contrasting habitat type, plant size, stomatal size and number, leaf shape, and leaf relative curling rates in Chloridoideae and Panicoideae, but differences in leaf hydraulic and gas exchange traits between the two subfamilies are weak. This pattern can be explained by the interactions of subfamily×C_4_ subtype and subfamily×habitat water availability in affecting ecophysiological traits, especially those linked to leaf water relations. Phylogeny and C_4_ subtype each tend to have stronger effects on structural and physiological traits, respectively, and the interaction between subfamily and habitat type results in different adaptation strategies. All Chloridoideae species have faster relative leaf curling rates than Panicoideae, irrespective of C_4_ subtype; but Chloridoideae species have two different ways to reach higher drought resistance potential than Panicoideae, NAD-ME species by drought avoidance, and PCK species by osmotic adjustment. This study elucidated the roles of C_4_ subtype and habitat type in affecting ecophysiological differences between subfamilies. The work expands our understanding of water relations in major C_4_ grass lineages, with implications for explaining their regional and global distributions in relation to climate.

## Supplementary data

Supplementary data are available at *JXB* online.

Table S1. Species list with seed source accessions, photosynthetic subtype, life form, and habitat type.

Table S2. PCA and phylogenetic PCA (PPCA) for the first two principal components based on 29 plant traits of 33 C_4_ grasses.

Table S3. Parameters for PV curves in subgroups in Fig. 4.

Figure S1. The consistency among MAP, water score and habitat type.

Figure S2. Phylogenetic relationships of the 33 species with C_4_ photosynthetic subtypes labelled.

Supplementary Data
