# Supplementary material for: Water relations traits of C4 grasses depend on phylogenetic lineage, photosynthetic pathway, and habitat water availability
Source: J Exp Bot. 2014 Dec 12;66(3):761–73. doi: 10.1093/jxb/eru430 (PMC4321540; doi:10.1093/jxb/eru430)
Supplement: Supplementary Data [file supp_66_3_761__index.html]

Water relations traits of C4 grasses depend on phylogenetic lineage, photosynthetic pathway, and habitat water availability — Water relations traits of C4 grasses depend on phylogenetic lineage, photosynthetic pathway, and habitat water availability — Supplementary Data 

# Water relations traits of C4 grasses depend on phylogenetic lineage, photosynthetic pathway, and habitat water availability

## Supplementary Data

Data files

**Files in this Data Supplement:**

- Supplementary Data - Supplementary Data
