# Supplementary material for: Water relations traits of C4 grasses depend on phylogenetic lineage, photosynthetic pathway, and habitat water availability
Source: J Exp Bot. 2014 Dec 12;66(3):761–73. doi: 10.1093/jxb/eru430 (PMC4321540; doi:10.1093/jxb/eru430)
Supplement: Supplementary Data [file supp_eru430_jexbot132845_file001.pdf]

Water relations traits of C_4 grasses depend on phylogenetic lineage, photosynthetic pathway and habitat water availability

Hui Liu, Colin P. Osborne

Supplementary data

Table S1 Information about the 33 species used in the glasshouse experiment. Annual or perennial categories based on Kew Grassbase (Clayton *et al*., 2006 onwards). “inter” in habitat type means “intermediate” species.

Clade	C_4 subtype	Species	Source[1]	Seed ID	Annual/ perennial	Habitat type
**Subfamily Panicoideae**						
Paspaleae	NADP-me	*Axonopus compressus*	A	PI406648	P	wet
	NADP-me	*Panicum antidotale*	B	CIAT6039	P	inter
	NADP-me	*Paspalum dilatatum*	A	PI477099	P	inter
Paniceae	NAD-me	*Alloteropsis cimicina*	C	AusTRCF 59560	A/P	inter
	PCK	*Alloteropsis semialata*	D		P	arid
	PCK	*Brachiaria decumbens*	C	AusTRCF 72793	P	inter
	PCK	*Brachiaria nigropedata*	C	AusTRCF 319409	P	arid
	NADP-me	*Digitaria eriantha*	C	AusTRCF 319398	P	inter
	PCK	*Melinis ambigua*	C	AusTRCF 52263	P	inter
	PCK	*Melinis repens*	E		A/P	inter
	NADP-me	*Pennisetum clandestinum*	C	AusTRCF 60072	P	wet
	NAD-me	*Panicum virgatum*	A	PI476292	P	inter
	NADP-me	*Setaria incrassata*	C	AusTRCF 24582	P	wet
	PCK	*Urochloa mosambicensis*	C	AusTRCF 320249	P	inter
	PCK	*Urochloa maxima*	A	PI259549	P	wet
Andropogoneae	NADP-me	*Andropogon gayanus*	C	AusTRCF24575	P	inter
	NADP-me	*Bothriochloa pertusa*	C	AusTRCF 106426	P	inter
	NADP-me	*Heteropogon contortus*	A	Grif 15979	P	inter
	NADP-me	*Imperata cylindrica*	E		P	inter
	NADP-me	*Ischaemum molle*	C	AusTRCF84176	P	inter
	NADP-me	*Arundinella hirta*	A	PI263693	P	wet
**Subfamily Chloridoideae**						
Cynodonteae	NAD-me	*Cynodon dactylon*	C	AusTRCF 37896	P	inter
	PCK	*Chloris elata*	C	AusTRCF 53926	P	inter
	PCK	*Chloris gayana*	C	AusTRCF 319691	P	wet
	PCK	*Chloris roxburghiana*	C	AusTRCF 59653	P	arid
	PCK	*Dactyloctenium aegyptum*	F	91323	A	arid

	PCK	*Dactyloctenium scindicum*	C	AusTRCF25452	P	arid
Eragrostideae	NAD-me	*Eragrostis curvula*	C	AusTRCF 30368	P	inter
	NAD-me	*Enneapogon scoparius*	C	AusTRCF59846	P	arid
	NAD-me	*Fingerhuthia africana*	C	AusTRCF98990	P	arid
Zoysieae	NAD-me	*Sporobolus nebulosus*	G	0082459	P	inter
	PCK	*Spartina pectinata*	D	30925	P	wet
	PCK	*Zoysia japonica*	F	91937	P	inter

[1]Suppliers: A, US Department of Agriculture National Plant Germplasm System (NPGS), BARC-West Beltsville, MD, USA; B, International Center for Tropical Agriculture (CIAT), Genetic Resources Program (GRP); C, Australian Plant Genetic Resource Information Service (AusPGRIS), Queensland, Australia; D, collected in South Africa, stored in the University of Sheffield; E, Silverhill seeds, Cape Town, South Africa; F, Herbiseed, Twyford, UK; G, grown in Tapton Experimental Gardens of the University of Sheffield by Taylor *et al.* (2010).

Table S2 (a) Conventional principal component analysis (PCA) and (b) phylogenetic PCA (PPCA) for the first two principal components based on 29 plant traits of 33 C_4 grasses. PC loadings and the percentage of variance explained by the first two PCs are reported. For each PC, the first seven variables with highest loadings are in bold. See trait abbreviations in Fig. 1.

**(a)** PCA			**(b)** PPCA		
Variable	PC1	PC2	Variable	PC1	PC2
LD	**-0.33**	0.07	LD	**-0.83**	0.19
LDMC	**-0.31**	0.10	LDMC	**-0.82**	0.24
LA	**-0.30**	-0.17	SLA	**0.66**	0.27
Height	**-0.28**	-0.15	Height	**-0.65**	-0.40
TLP	**-0.28**	0.12	WUE_i	**-0.62**	0.46
SLA	**0.27**	0.14	LA	**-0.62**	**-0.62**
ε	**-0.26**	0.10	TLP	**-0.60**	0.22
GL	0.06	**-0.33**	LV	-0.53	**-0.71**
SW	0.03	**-0.32**	LT	0.21	**-0.59**
Seed	-0.04	**-0.29**	LW	-0.46	**-0.56**
LT	0.06	**-0.28**	K_{leaf}	0.46	**-0.56**
LW	-0.20	**-0.26**	g_s	0.55	**-0.54**
X1	0.12	**0.26**	Ψ_{sat}	0.23	**0.51**
SD	-0.18	**0.23**			
Total variance %	23%	18%		24%	16%
Cumulative variance %	23%	41%		24%	40%

Table S3 Parameters derived from PV curves for different C_4 subtypes of two subfamilies in Figure 3. The highest and lowest values for each index are in bold. Abbreviations are the same as in Table 1.

	n	Ψ_{sat} (MPa)	Ψ_{osat} (MPa)	TLP (MPa)	RWC at TLP (%)	ε (MPa)
Chloridoideae						
NAD-me	25	**-0.197**	-0.937	-1.048	**90.68**	**0.097**
PCK	35	-0.195	**-1.152**	**-1.316**	91.54	**0.139**
Panicoideae						
NAD-me	10	**-0.152**	**-0.802**	**-0.892**	91.62	0.100
NADP-me	60	-0.159	-1.052	-1.193	**91.72**	0.110
PCK	35	-0.153	-0.972	-1.101	91.45	0.109

Figure legends

Fig. S1. (a) Relationship between MAP and water score, and (b) distribution of MAP for each habitat type for the 33 grass species used in the greenhouse experiment. Subfamilies are Chloridoideae (black) and Panicoideae (grey). Regression line in (a): $y = 193 \times x + 488$ ($R^2 = 0.11$***).

Fig. S2. Phylogenetic relationships of the 33 species used in the greenhouse experiment. Subfamilies and lineages are labelled. C_4 photosynthetic subtypes (PT) are shown in different shapes: PCK (triangle), NAD-me (square) and NADP-me (round).

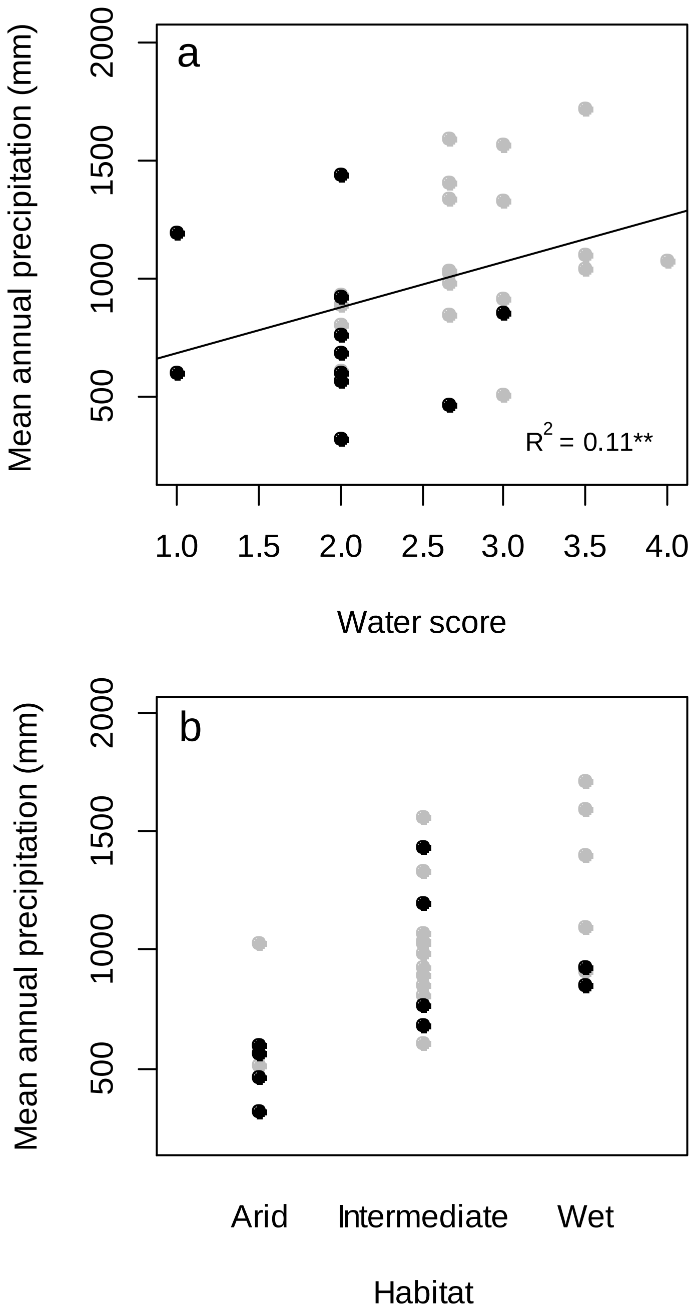

Fig. S1. Liu *et al.*

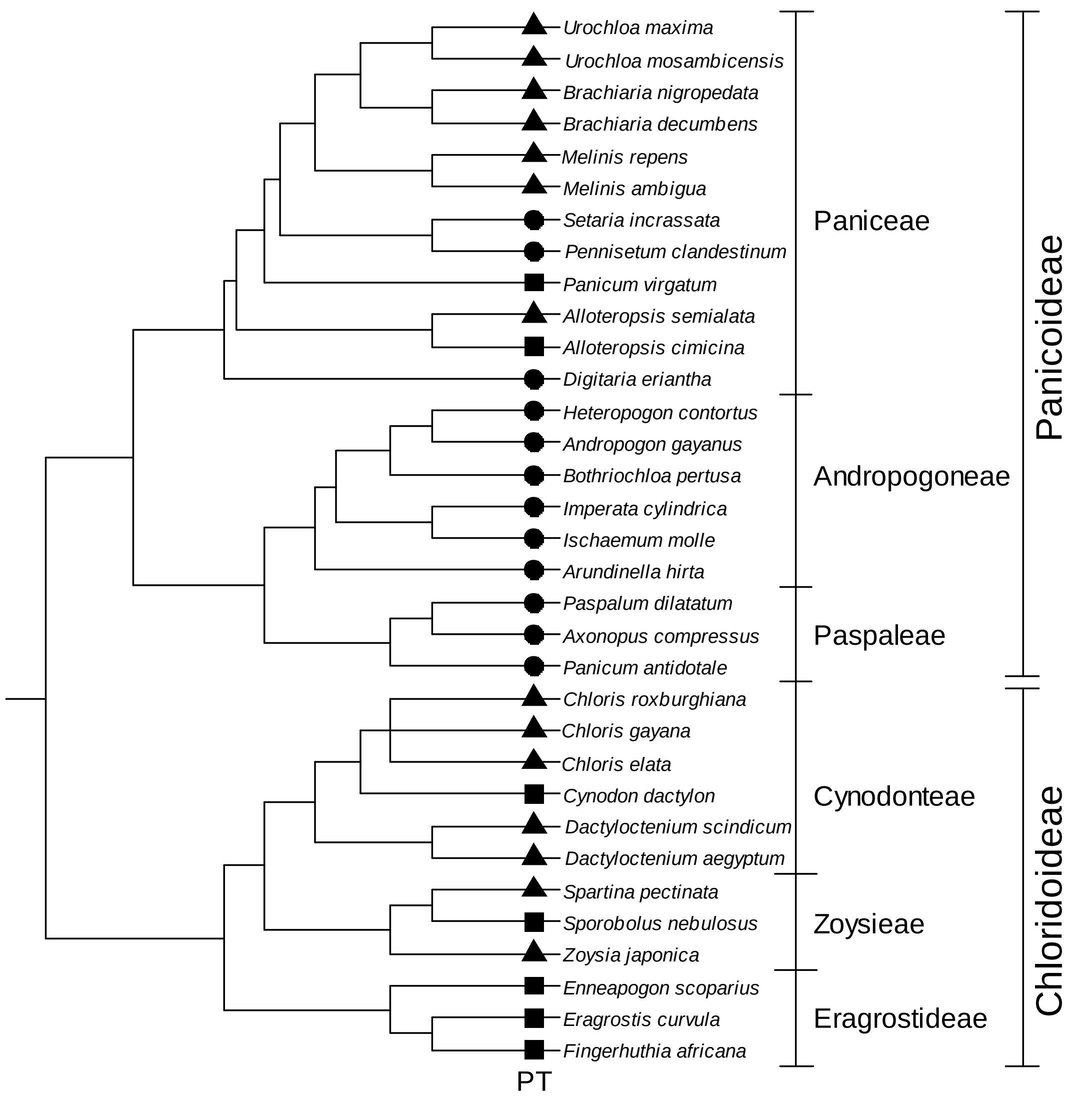

Fig. S2. Liu *et al.*
